# Pycnogenol's Dual Impact: Inducing Apoptosis and Suppressing Migration via Vascular Endothelial Growth Factor/Fibroblast Growth Factor Signaling Pathways in Breast Cancer Cells

**DOI:** 10.7759/cureus.65286

**Published:** 2024-07-24

**Authors:** Shreyas Somesh, Jeevitha Rajanathadurai, Elumalai Perumal

**Affiliations:** 1 Center for Global Health Research, Saveetha Medical College and Hospitals, Saveetha Institute of Medical and Technical Sciences, Chennai, IND; 2 Cancer Genomics Laboratory, Center for Global Health Research, Saveetha Medical College and Hospitals, Saveetha Institute of Medical and Technical Sciences, Chennai, IND

**Keywords:** in-vitro phytochemicals, cell migration, apoptosis, brest cancer, pycnogenol, anti-cancer drug discovery

## Abstract

Background:The leading cause of cancer-related fatalities in women globally is breast cancer. Chemotherapy is one of the traditional therapies for breast cancer, even though it does not target cancer cells directly and has major side effects. As a result, the development of novel therapeutic techniques with improved safety and effectiveness is constantly required.

Aim:* *This study aimed to investigate the pro-apoptotic and anti-migrative effects of pycnogenol in a breast cancer cell line.

Methodology:* *By using the 3-[4,5-dimethylthiazol-2-yl]-2,5 diphenyl tetrazolium bromide (MTT) method, the cell viability of breast cancer cells treated with pycnogenol was evaluated. Pycnogenol was applied to the MCF-7 cells in a range of concentrations (20-120 µg/ml) for 24 hours. A phase contrast microscope is used to evaluate changes in cell morphology. In breast cancer cells, acridine orange (AO) and ethidium bromide (EtBr) dual staining were employed to analyze the nuclear morphological alterations. A fluorescent microscope was used to see the apoptotic nuclei. A scratch wound healing assay was performed to evaluate the anti-migrative potential of pycnogenol. Gene expression analysis was performed using quantitative real-time PCR to determine the levels of proapoptotic and vascular endothelial growth factor (VEGF)/VEGF receptor (VEGFR) genes mRNA expression.

Results: In our investigation, breast cancer cells treated with pycnogenol displayed a substantial reduction in cell viability and a statistically significant p<0.05 between the control and treatment groups. We observed inhibitory concentrations (IC-50) at 80 μg/mL in breast cancer cells. After treatment, fewer cells were present, and those that were there shrank and showed cytoplasmic membrane blebbing. Under AO/EtBr staining, treated cells show chromatin condensation and nuclear fragmentation. The results of this study revealed a significant downregulation of Bcl-2, VEGF/FGF, and p53 mRNA expression following treatment with pycnogenol. Furthermore, the impact of pycnogenol on cell migration decreased significantly when compared to control cells. Pycnogenol treatment significantly induces apoptosis and inhibits migration by altering the VEGF signaling pathway.

Conclusion:* *Overall, this study highlights the promising role of pycnogenol as a proapoptotic and antimigrative agent through the inhibition of anti-apoptotic and VEGF/FGF signaling molecules gene expression, offering new prospects for improving breast cancer treatment.

## Introduction

Breast cancer is the most prevalent cancer in women worldwide and the main cause of women's cancer-related fatalities [1]. Despite advances in early detection and treatment, breast cancer remains a major health concern. Breast cancer is a heterogeneous disease, and the subtypes differ in their molecular characteristics and treatment responses. Estrogen receptor-positive (ER+) breast cancer, which accounts for approximately 70% of all occurrences of breast cancer, is a surrogate marker of luminal A and luminal B, two of the most prevalent molecular subtypes of ductal breast carcinoma [2-4]. Endocrine therapy resistance is a major challenge in the treatment of ER+ breast cancer, and a significant fraction of ER+ breast cancer may benefit from concurrent chemotherapy (especially luminal B-type breast cancers); hence, there is a need for new treatment options [5-7]. Natural compounds have been explored as potential anti-cancer agents due to their ability to target multiple pathways in cancer cells while having minimal toxicity to normal cell therapies [8]. One such compound is pycnogenol, a natural extract obtained from the bark of the French maritime pine tree (Pinus pinaster) [6,9]. It is well known for having high concentrations of catechins, proanthocyanidins, taxifolin, and other polyphenolic chemicals, which are examples of natural bioflavonoids [10]. Strong antioxidants, these bioflavonoids have been linked to several health advantages, including anti-cancer properties. Research on their specific anti-cancer activity is still in its early stages. Some studies suggest that the bioflavonoids present in pycnogenol may have potential anti-cancer effects by inhibiting cell proliferation, inducing apoptosis, and preventing angiogenesis. 

In recent years, natural compounds such as pycnogenol have been explored as potential alternatives or complementary therapies for cancer [11,12]. Pycnogenol is a potent antioxidant that helps neutralize free radicals in the body, which can cause oxidative stress and damage cells [13]. Pycnogenol has been shown to possess various pharmacological properties, including antioxidant, anti-inflammatory, and anti-cancer effects. Previous studies have demonstrated that pycnogenol induces apoptosis in various cancer cell lines, including breast cancer. However, the mechanisms by which pycnogenol induces apoptosis in breast cancer cells are not fully understood. Previous studies suggest that pycnogenol may influence cellular signaling pathways related to cell growth, survival, and apoptosis (programmed cell death). According to earlier research, pycnogenol therapy caused apoptosis and slowed the proliferation of cancer cells. The previous study suggests that the suppression of the Akt signaling pathway could be implicated in pycnogenol's anti-cancer effect by inhibiting cell proliferation and inducing apoptosis. Pycnogenol was examined in a different investigation to see how it affected human myeloid leukemia cells. The researchers saw that treating these cancer cells with pycnogenol caused apoptosis and slowed down their proliferation. The study also demonstrated how caspases and reactive oxygen species contribute to pycnogenol's anti-cancer properties [14]. Pycnogenol has been shown to have potent antioxidant and anti-inflammatory properties, and several studies have demonstrated its potential to suppress the growth of cancer cells in vitro and in vivo [15].

While these earlier studies offer some information about pycnogenol's possible anti-cancer properties, it's important to remember that the majority of the studies have been done using in vitro models. Understanding the complex web of signaling channels that controls the process of apoptosis, which is a type of planned cell death and cancer metastasis, is very important for cancer management. Angiogenesis, the formation of new blood vessels from pre-existing ones, is a crucial process in the growth and progression of breast cancer. Tumor cells secrete various angiogenic factors, including vascular endothelial growth factor (VEGF) and fibroblast growth factor (FGF), which promote angiogenesis and tumor growth. Therefore, targeting angiogenesis and the VEGF/FGF signaling pathway is an attractive therapeutic approach for breast cancer treatment [16]. Cancer is characterized by dysregulation of apoptosis, and methods to cause apoptosis in cancer cells have been investigated as potential cancer treatments [17]. Apoptosis, a programmed cell death process, plays an essential role in maintaining tissue homeostasis and preventing the growth of cancer cells [18]. Apoptosis dysregulation is a hallmark of cancer, and defects in this process contribute to cancer development and progression [19]. Therefore, induction of apoptosis in cancer cells is an attractive therapeutic strategy for cancer treatment. Anti-apoptotic genes, such as B-cell lymphoma 2 (Bcl-2) and Bcl-extra-large (Bcl-xL), play a critical role in regulating the apoptotic process. Overexpression of these genes has been shown to confer resistance to apoptosis and contribute to cancer progression. Therefore, targeting these proto-oncogenes, such as by suppressing expression (i.e., via promoter methylation), reducing RNA stability (via shRNA), or promoting protein degradation (i.e., via ubiquitination), is an attractive strategy [19]. In this study, we examined pycnogenol's cytotoxic and pro-apoptotic effects on the breast cancer cell line MCF-7. We have also studied the antimigrative potential of pycnogenol in breast cancer cells by targeting the VEGF signaling. A popular breast tumor cell line in research is MCF-7, which is an ER+ breast cancer cell line (MCF-7) used to evaluate pycnogenol's cytotoxic and pro-apoptotic effects.

## Materials and methods

Cell line maintenance

The National Centre for Cell Science (NCCS), Pune, provided breast cancer cell lines (MCF-7) for this study. The cells were grown in T25 culture flasks using 10% fetal bovine serum (FBS) and 1% antibiotics in addition to Eagle's Medium, Modified with Dulbecco (DMEM). The cells were maintained at 37°C in a moist atmosphere with 5% CO2. After being sterilized, confluent cells were passaged.

Cell viability (MTT) assay

To determine the survival rate of pycnogenol-treated carcinoma of the breast cells, the MTT test was used. The MTT (3-[4,5-dimethylthiazol-2-yl]-2,5-diphenyl tetrazolium bromide) assay measures how well cells that are metabolically engaged transform tetrazolium salt into formazan crystals. The cells were grown in 96-well plates with a 5x103 cell density per well. Cells were starved for three hours at 37°C in serum-free media after being washed twice with 100 µl of serum-free medium 24 hours after plating. Cells were incubated with varying doses (20-120 µg/ml) of pycnogenol for 24 hours after a fast. The medium from the control and treated cells were put into each well along with 100 µl of MTT composed of DMEM (0.5 mg/ml) following treatment. Following that, the cells were maintained at 37° in the CO2 incubator for 4 hours. After discarding the MTT-containing medium, the created formazan crystals were mixed with dimethyl sulfoxide (100µl), and a MicroELISA plate reader was used to measure the color intensity at 570 nm. As a percentage of control cells grown in serum-free media, cell viability was calculated. Cell viability in the control, untreated medium was estimated using the following formula and represented as 100%: The formula for percentage cell viability is [A570 nm of treated cells/A570 nm of control cells] x 100 [20].

Morphology study

We chose the best doses (IC-50: 80 ug/ml) for further research based on the MTT assay. A phase-contrast microscope is used to examine changes in cell morphology. In six-well plates, 2x105 cells were plated and given a treatment for cancer cells at a concentration of 20 ug/ml for 24 hours. The medium that was used was taken out of the cells after the time of incubation, and they were given a single rinse in phosphate-buffered saline (PBS, pH 7.4). With the use of a phase contrast microscope, the plates were examined.

Double labeling using the orange dye acridine (AO) and a substance called ethidium bromide (EtBr) to determine the manner of cell death

By using AO/EtBr dual staining, it was also possible to assess how a medicine affected cancer cell death. The cells were given the medication (20 ug) for 24 hours, after which the cells were extracted and cleaned with ice-cold PBS (phosphate-buffered saline). The granules were immersed in 5 mL each of acridine orange and ethidium bromide (EtBr), each at a concentration of 1 mg/mL. A fluorescent microscope was then used to view the labeled cells' apoptotic changes.

Gene expression analysis by real-time PCR

The gene expression of apoptotic and VEGF/FGF genes was analyzed using real-time PCR. The total RNA was isolated by the standardized protocol using Trizol Reagent (Sigma). 2μg of RNA was used for cDNA synthesis using reverse transcription using a PrimeScript 1st strand cDNA synthesis kit (TakaRa, Japan). The targeted genes were amplified using specific primers P53: F- “AGGCCTTGGAACTCAAGGAT” R-“TGAGTCAGGCCCTTCTGTCT”; Bcl2: F- “CATGTGTGTGGAGAGCGTCAAC” R-”CAGATAGGCACCCAGGGTGAT”; GAPDH: F-“CGACCACTTTGTCAAGCTCA” R-“CCCCTCTTCAAGGGGTCTAC”. The PCR reaction was performed with GoTaq® qPCR Master Mix (Promega), which contains SYBR green dye and all the PCR components. Real-time PCR was performed on a CFX96 PCR system (Biorad). The results were analyzed by the comparative CT method, and the 2−∆∆CT method was used for the fold change calculation described by Schmittgen and Livak [21].

Cell migration analyzed by scratch wound healing assay

Human breast cancer cell lines (2×105 cells/well) were seeded onto six-well culture plates. The cell monolayer was scratched using a 200μl tip to create a wound. The detached cells were removed by washing with 1X PBS and adding fresh culture medium with 80 μg/ml of pycnogenol for 24 h, along with the control group. After incubation, the wells were washed and fixed in 4% paraformaldehyde. Photographs were taken using an inverted microscope (Euromex, The Netherlands).

Statistical analysis

All data were acquired, reported as mean SD for triplicates, and analyzed using one-way ANOVA and a Student's t-test in SPSS. A p< 0.05 cutoff was used to determine statistical significance.

## Results

The cytotoxic and pro-apoptotic potential of pycnogenol on the MCF-7 carcinoma of the breast cell line

**Figure 1:**
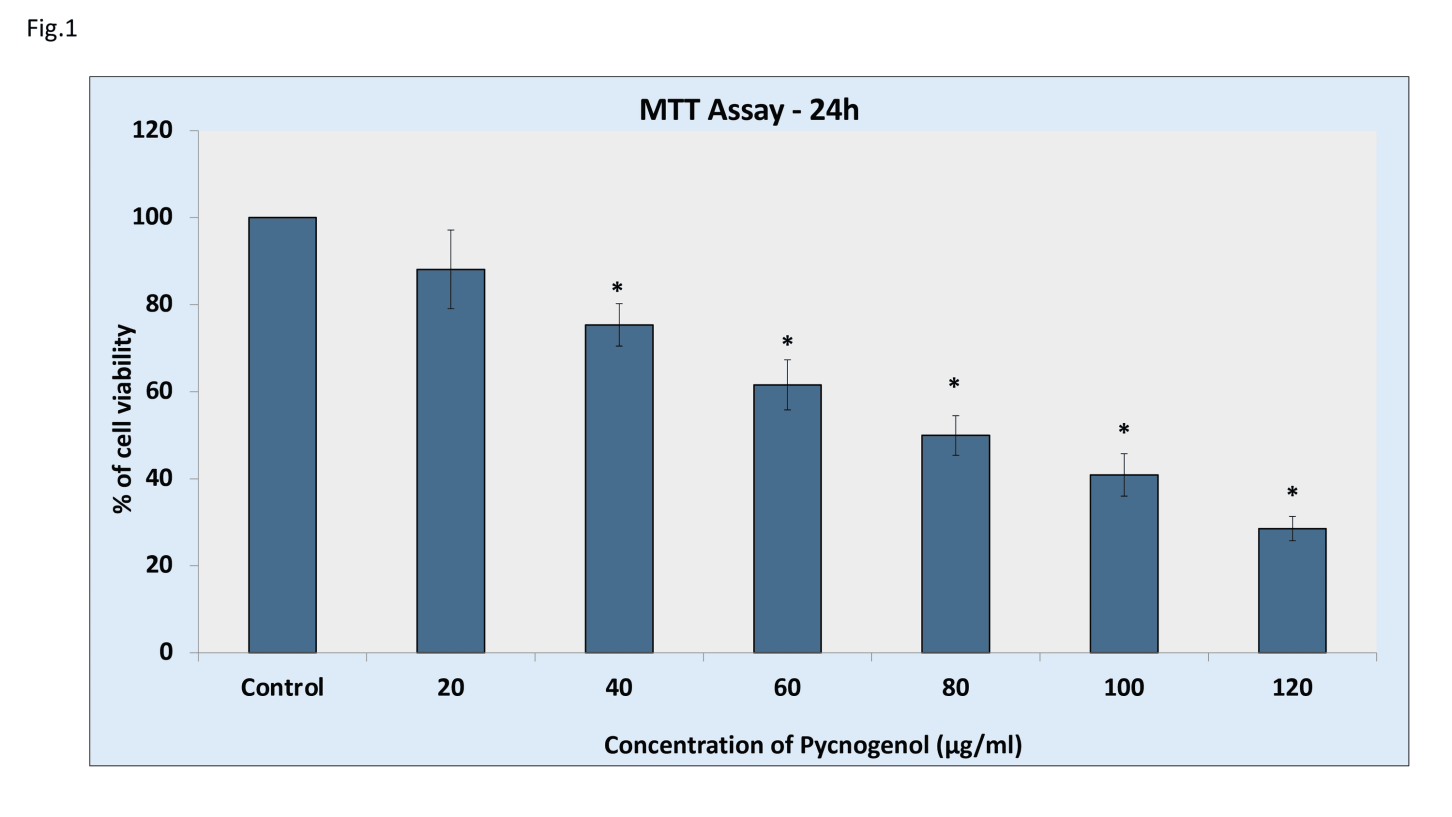
Cytotoxic and pro-apoptotic potential of pycnogenol on the MCF-7 carcinoma of the breast cell line The cytotoxic impacts of pycnogenol on carcinoma of the breast cells. Pycnogenol (20-120 µg/ml) was applied to the cells for 24 hours, and the MTT test was used to determine the vitality of the cells. Treatment with pycnogenol drastically decreases the survival rate of breast cancer cells in a dose-dependent way. Data are presented as mean standard deviations (n = 3); * when compared to the control blank group, p< 0.05.

Various concentrations (20-120 µg/ml) were applied to the cells for a total of 24 hours. Our study discovered that, compared to the control, stimulation with Pycnogenol tree extract significantly decreased the sustainability of MCF-7 breast tumor cells at the 24-hour time point (Figure 1). Increased concentration led to a growing decrease in the number of viable cells. At a dosage of 80 µg/ml, we observed a 50% growth inhibition, which was recognized as the inhibition level (IC-50) dosage value and taken into account for the following research.

**Figure 2:**
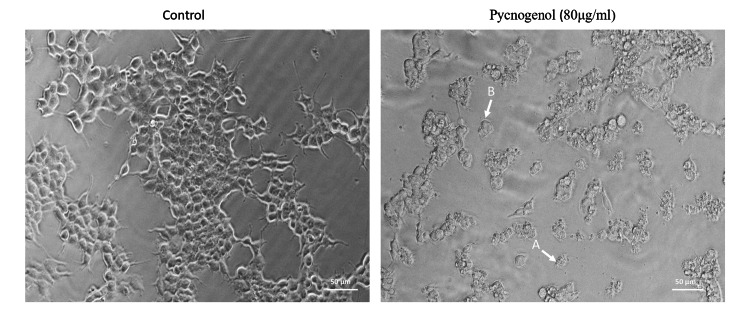
Effect of pycnogenol on the cell morphology of human breast cancer (MCF-7) The cells were treated with pycnogenol (80 ug/ml) for 24 hours, and cells were observed under inverted phase contrast microscope. The number of cells decreased after pycnogenol treatment, and the cells exhibited cell shrinkage and cytoplasmic membrane blebbing.

In comparison to the untreated cells, the MCF-7 breast cancer cell line underwent a 24-hour treatment with 80 µg/ml of pycnogenol extract and showed significant morphological changes (Figure 2). Cell atrophy and diminished cell count, both indicators of apoptotic cells, were among these modifications. In addition, cells experiencing death displayed additional changes in shape, such as rounder cells that shrank and lacked a connection to neighboring cells. A few delicate cells were also separated from the plates' surface.

**Figure 3:**
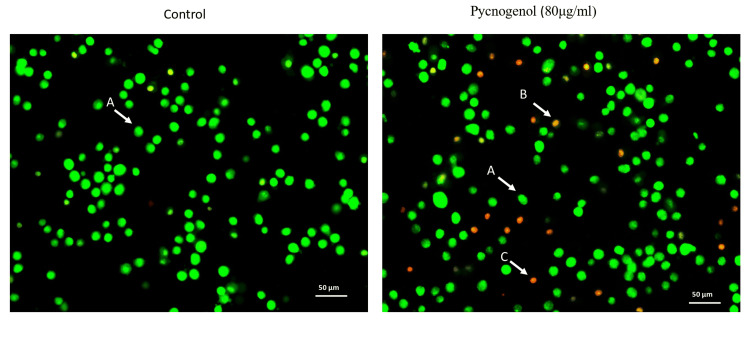
Detection of apoptotic cells in pycnogenol-treated breast cancer cells by AO/ETBr dual staining Human breast cancer cells were treated with pycnogenol (80 µg/ml) for 24 h, along with the control group. After the treatment, the cells were incubated with AO/EtBr dual staining. Fluorescence images were captured using an inverted fluorescence microscope (20X). Viable cells (A) are characterized by a uniformly bright green nucleus; early apoptotic cells (B) exhibit bright orange areas of condensed or fragmented chromatin in the nucleus; and late apoptotic cells (C) display a uniformly bright red nucleus. These observations were consistent across at least three independent experiments with similar parameters.

**Figure 4:**
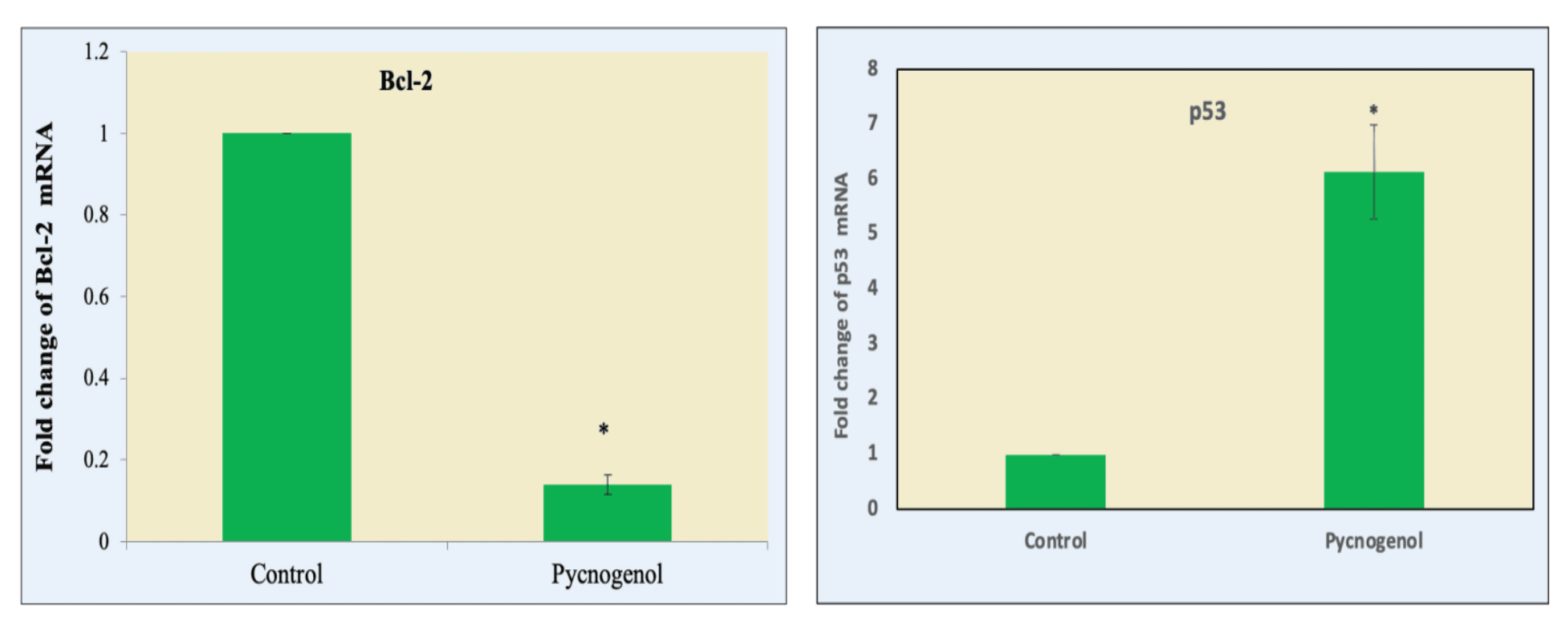
Effect of pycnogenol (80 μg/ml for 24 h) on Bcl-2 and p53 gene expression in breast cancer cell lines Target gene expression is normalized to GAPDH mRNA expression, and the results are expressed as a fold change from the control. Each bar represents the mean + SEM of three independent observations. ‘*’ represents statistical significance between control and drug treatment groups at the p<0.05 level.

Following a 24-hour exposure to a pyrogenol sample (80 µg/ml), the nuclear appearance of apoptotic cells is investigated using AO/EtBr dual-labeling. Fluorescence microscopy was used to observe the treated cells after they had been stained with AO/EtBr dye for 20 minutes. The observed outcomes showed that although EtBr exclusively marked cells that had lost the strength of their membrane, AO marked equally alive and dying cells. Yellow- and orange-stained cells are in the beginning and end stages of apoptosis, respectively, whereas green-stained cells are still alive. In the current investigation, control cells displayed a consistent green hue, but cells treated with pycnogenol extract displayed yellow, orange, and red signals (Figure 3). These findings demonstrate that pycnogenol extract promotes apoptosis in MCF-7 breast cancer cells. Bcl-2 and p53 genes are considered markers of MCF-7 breast cancer cells. MCF-7 cells are used ubiquitously in research for ER-positive breast cancer cell experiments, with the majority of the investigations into acquired anti-estrogen drug resistance having utilized them. Anti-apoptosis is thought to be a crucial step in cancer cell metastasis, as it enables cancer cells to gain migratory and invasive capabilities. We observed that pycnogenolegulation of anti-apoptotic genes was associated with the inhibition of breast cancer cell migration. Treatment significantly inhibited Bcl-2 and p53 anti-apoptotic gene expression in breast cancer cells (Figure 4). This downregulation of anti-apoptotic genes was associated with the inhibition of breast cancer cell migration.

Pycnogenol inhibits the migratory potential of breast cancer cells by inhibiting the VEGF/FGF signaling pathway.

**Figure 5:**
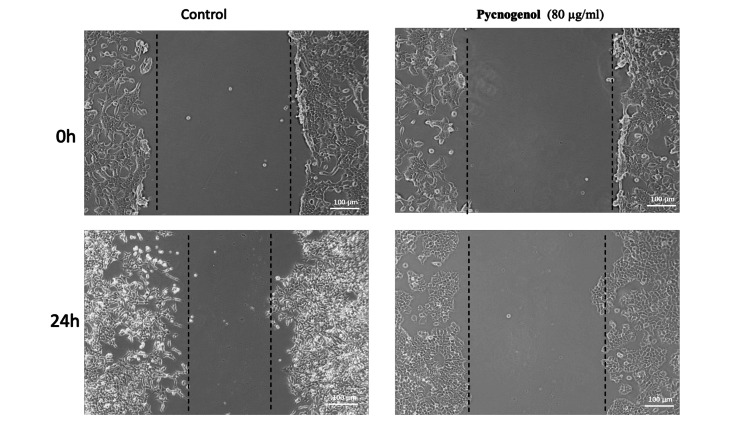
The anti-migrative potential of pycnogenol against breast cancer cells was assessed by an in vitro scratch wound healing assay Human breast cancer cells were injured, and a cell migration assay with and without treatment of pycnogenol (80 µg/ml) was performed at 24 h. Images were obtained using an inverted phase contrast microscope.

Cancer metastasis is characterized by the migration of cells from one site to another, which plays a crucial role in tumor invasion and spread. This migratory capacity is acquired by cancer cells through alterations in cytoskeletal dynamics and adhesion properties, enabling them to detach from the primary tumor and invade adjacent tissues. Investigating metastasis and cell migration in a controlled cell culture environment is instrumental in identifying potential therapeutic targets and devising innovative anti-cancer approaches. A scratch test was performed to evaluate the effect of pycnogenol on the migration of breast cancer cells. The results showed that pycnogenol inhibits the cell migration rate when compared to control cells. The following observations were made: In the control group, untreated cancer cells migrated to almost half of the scratched area after 24 hours. Treatment with pycnogenol at a concentration of 80 μg/mL significantly inhibited the migration of breast cancer cells compared to the control group. The migration distance of cells in the pycnogenol group decreased compared with that observed in the control group (Figure 5). 

**Figure 6:**
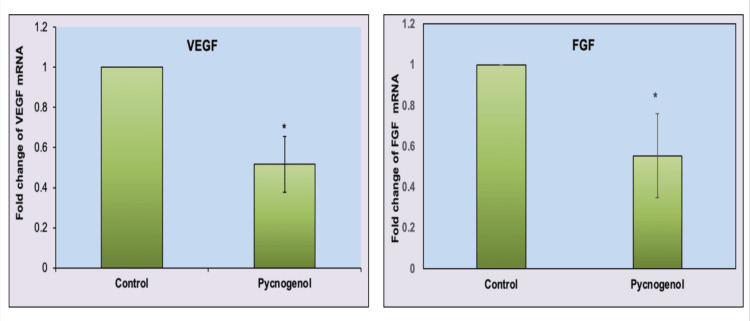
Effect of pycnogenol (80 μg/ml) on VEGF/FGF gene expression in the breast cancer cell line (MCF-7) Target gene expression is normalized to GAPDH mRNA expression, and the results are expressed as a fold change from the control.  Each bar represents the mean + SEM of three independent observations. ‘*’ represents statistical significance between control and drug treatment groups at the p<0.05 level.

Subsequently, we investigated the mRNA expression of VEGF-A/FGF-2 signaling molecules in breast cancer cells. The vascular endothelial growth factor-A (VEGF-A) and fibroblast growth factor-2 (FGF-2) signaling pathways play significant roles in breast cancer development and progression. VEGF is a key regulator of angiogenesis, promoting the formation of new blood vessels to supply nutrients and oxygen to tumors. Elevated levels of VEGF are associated with increased angiogenesis and are often observed in breast cancer tissues. Similarly, FGF signaling is implicated in breast cancer progression. Aberrant activation of VEGF and FGF signaling can contribute to tumor growth and metastasis in breast cancer. Therapeutic approaches targeting VEGF/FGF receptors or downstream signaling components are being explored to impede cancer progression. We employed real-time PCR to assess the mRNA expression of VEGF-A/FGF-2 in MCF-7 cell lines. We observed that pycnogenol treatment significantly inhibited VEGF-A/FGF-2 gene expression in breast cancer cells. This downregulation of VEGF-A/FGF-2 was associated with the inhibition of breast cancer cell migration (Figure 6).

## Discussion

This research investigates the multifaceted effects of pycnogenol on breast cancer cells, specifically focusing on its pro-apoptotic and anti-migrative properties through the modulation of p53/Bcl-2 and VEGF/FGF signaling pathways. The study delves into the intricate molecular mechanisms underlying these effects, shedding light on the potential therapeutic implications of pycnogenol in breast cancer management. The pro-apoptotic role of pycnogenol is explored in detail, emphasizing its ability to induce programmed cell death in breast cancer cells. The study employs various molecular assays to assess apoptosis-related markers such as p53 activation and changes in Bcl-2 family proteins. These findings provide crucial insights into the apoptotic pathways influenced by pycnogenol, contributing to a deeper understanding of its anticancer properties.

In this study, we looked into pycnogenol's cytotoxic and pro-apoptotic effects on the breast cancer cell line MCF-7. Initially, the breast cancer cell line was subjected to varying concentrations of pycnogenol (20-120 μg/ml) for 24 hours to evaluate its inhibitory effect on the growth of breast cancer cells. Our findings demonstrated that pycnogenol administration significantly reduced MCF-7 cells' viability in a dose- and time-dependent manner. Pycnogenol significantly caused cell death at higher concentrations (80 µg/ml), pointing to a potential cytotoxic action. The IC50 value of 80 μg/ml was selected to further assess the inhibitory impact, and the morphology was examined using a phase-contrast microscope to confirm its anticancer potential. Here, the breast cancer cells were significantly reduced after treatment with pycnogenol for 24 hours, and the cells exhibited indications of cytotoxicity by shrinking and blebbing of the cytoplasmic membrane.

Apoptosis, also known as programmed cell death, is distinguished by DNA fragmentation, cell shrinkage, chromatin condensation, and the activation of certain enzymes known as caspases [22]. The progression of cancer arrests the apoptotic process. The most well-established anticancer strategy involves inducing apoptosis in tumor cells, and it is used in numerous cancer treatments [21-23]. Our results showed that pycnogenol treatment significantly increased the percentage of apoptotic cells, indicating a pro-apoptotic effect. AO/EtBr dual staining was performed to confirm the pycnogenol-induced apoptotic cells in breast cancer cells. Human promyeloid leukemia cells were used in the prior work to investigate pycnogenol's effects. The leukemia cells' death and cell differentiation were both induced by the pycnogenol therapy, according to the findings. Pycnogenol may provide therapeutic benefits for treating leukemia, according to the study [14]. However, previous studies have suggested that pycnogenol may exert its effects by modulating the signaling pathways involved in cell survival and apoptosis [11]. For example, pycnogenol has been shown to activate the MAPK/ERK pathway, which plays a crucial role in regulating cell proliferation, differentiation, and apoptosis [24,25].

Furthermore, the research elucidates the anti-migrative potential of pycnogenol, particularly its impact on VEGF/FGF signaling pathways. Migration is a critical aspect of cancer progression, and the study investigates how pycnogenol may interfere with key factors involved in this process. By examining the expression levels of VEGF and FGF, the study establishes a link between pycnogenol treatment and the inhibition of migratory capabilities in breast cancer cells. The findings of this study shed light on the inhibitory effects of pycnogenol on VEGF and FGF mRNA expression in breast cancer cells. VEGF and FGF are crucial signaling molecules implicated in angiogenesis, a process essential for tumor growth and metastasis. The downregulation of VEGF and FGF is of particular significance in cancer research, as these molecules play pivotal roles in promoting the formation of new blood vessels and supplying nutrients to the growing tumor. Inhibiting their expression can impede angiogenesis, presenting a promising avenue for anticancer therapy. The results demonstrated a significant reduction in the mRNA expression of both VEGF and FGF, indicating the potential of pycnogenol to interfere with key pathways involved in angiogenesis. The dual action of inducing apoptosis and suppressing migration highlights the comprehensive nature of pycnogenol's impact on breast cancer cells. The study contributes valuable insights into the molecular mechanisms through which pycnogenol exerts its anticancer effects, specifically by targeting angiogenesis-related signaling pathways. These findings underscore the potential of pycnogenol as a therapeutic agent in breast cancer treatment, warranting further exploration and clinical investigation. 

In vitro anticancer activity studies of pycnogenol have limitations; these studies often fail to replicate the complexities of the human body's environment, including interactions with the immune system and various organs. Consequently, promising results observed in vitro may not accurately predict the efficacy and safety of pycnogenol when used in living organisms. For a more comprehensive understanding of the therapeutic potential of pycnogenol, further research involving understanding the molecular mechanism of anticancer activity and in vivo studies is essential to bridge the gap between laboratory findings and real-world application in cancer treatment.

## Conclusions

In conclusion, the research provides a comprehensive exploration of pycnogenol's effects on breast cancer cells, emphasizing its pro-apoptotic and anti-migrative activities mediated through the modulation of p53/Bcl-2 and VEGF/FGF signaling pathways. Pycnogenols demonstrated a cytotoxic impact on breast cancer cells that were concentration-dependent, inhibiting migration and viability of the cells while inducing apoptosis. Furthermore, the observed suppression of migration in breast cancer cells by pycnogenol underscores its potential role in mitigating metastasis, a critical factor influencing the prognosis of cancer patients. The interference with VEGF/FGF signaling pathways provides mechanistic insights into the anti-migratory effects of pycnogenol, suggesting its potential as a promising agent for preventing or limiting the spread of breast cancer. The findings contribute to the growing body of knowledge on natural compounds as potential adjuncts in cancer therapy and pave the way for further investigations into the clinical applications of pycnogenol in breast cancer treatment.
